# Characterizing the sublethal effects of SmartStax PRO dietary exposure on life history traits of the western corn rootworm, *Diabrotica virgifera virgifera* LeConte

**DOI:** 10.1371/journal.pone.0268902

**Published:** 2022-05-25

**Authors:** Jordan D. Reinders, Emily E. Reinders, Emily A. Robinson, William J. Moar, Paula A. Price, Graham P. Head, Lance J. Meinke

**Affiliations:** 1 Department of Entomology, University of Nebraska, Lincoln, Nebraska, United States of America; 2 Department of Statistics, University of Nebraska, Lincoln, Nebraska, United States of America; 3 CropScience Division, Bayer AG, Chesterfield, Missouri, United States of America; University of Tennessee, UNITED STATES

## Abstract

The western corn rootworm (WCR), *Diabrotica virgifera virgifera* LeConte, is an economically important pest of field corn (*Zea mays* L.) across the United States (U.S.) Corn Belt. Repeated use of transgenic hybrids expressing *Bacillus thuringiensis* (Bt) proteins has selected for field-evolved resistance to all current rootworm-active Bt proteins. The newest product available for WCR management is SmartStax^®^ PRO, a rootworm-active pyramid containing Cry3Bb1, Cry34/35Ab1 [now reclassified as Gpp34Ab1/Tpp35Ab1] and a new mode of action, DvSnf7 dsRNA. Understanding the fitness of adult WCR after dietary exposure to SmartStax^®^ PRO will identify potential impacts on WCR population dynamics and inform efforts to optimize resistance management strategies. Therefore, the objective of the present study was to characterize the effect of SmartStax^®^ PRO dietary exposure on WCR life history traits. Adult WCR were collected during 2018 and 2019 from emergence tents placed over replicated field plots of SmartStax^®^ PRO or non-rootworm Bt corn at a site with a history of rootworm-Bt trait use and suspected resistance to Cry3Bb1 and Cry34/35Ab1. Adult survival was reduced by 97.1–99.7% in SmartStax^®^ PRO plots relative to the non-rootworm Bt corn plots during the study. Individual male/female pairs were fed different diets of ear tissue to simulate lifetime or adult exposure. Life history parameters measured included adult longevity, adult head capsule width, lifetime female egg production, and egg viability. Results indicate that lifetime or adult exposure to SmartStax^®^ PRO significantly reduced adult longevity and lifetime egg production. Larval exposure to SmartStax^®^ PRO significantly reduced WCR adult size. Results from this study collectively suggest that SmartStax^®^ PRO may negatively impact WCR life history traits, which may lead to reduced population growth when deployed in an area with WCR resistance to Bt traits.

## Introduction

Transgenic field corn (*Zea mays* L.) hybrids expressing insecticidal proteins derived from *Bacillus thuringiensis* Berliner (Bt) have been used to manage western corn rootworm (WCR; *Diabrotica virgifera virgifera* LeConte) populations across the United States (U.S.) Corn Belt for almost two decades. Three rootworm-active Bt proteins were commercialized and marketed as single-trait products between 2003 and 2006: Cry3Bb1 [1], Cry34/35Ab1 (now reclassified as Gpp34Ab1/Tpp35Ab1) [2, 3], and mCry3A [4]. Continuous planting of single-protein Bt corn hybrids led to increasing reports of greater than expected root injury and subsequent confirmation of field-evolved resistance to Cry3 proteins [5–9] and Cry34/35Ab1 [8, 10] in multiple states across the U.S. Corn Belt. Varying levels of cross-resistance have also been documented between Cry3Bb1 and mCry3A [5, 7, 11, 12].

To improve insect resistance management (IRM), corn hybrids expressing two or more rootworm-active Bt proteins, defined as ‘pyramids’ [13, 14], have been introduced into the market to gradually replace single-protein hybrids [15]. Because all commercial rootworm-Bt pyramids contain at least one Bt protein originally sold as a single-trait product, the IRM value and durability of the pyramid may be reduced in areas with WCR field-evolved resistance to one or more Bt proteins. A fourth rootworm-active Bt protein, eCry3.1Ab, was registered in 2012 [16] and is only sold as a component of a rootworm-active pyramid containing mCry3A [17]. However, this protein has been shown to be cross-resistant with the other Cry3 proteins [11, 18]. Therefore, published data confirm all commercially available Bt proteins targeting the WCR are compromised to some degree in areas of the U.S. Corn Belt, highlighting the importance of developing additional products with novel modes of action to complement current tactics in WCR management programs.

SmartStax^®^ is a widely used Bt pyramid for WCR management that was commercialized in 2009 [19]. This transgenic pyramid contains Bt proteins targeting both lepidopteran (Cry1A.105, Cry2Ab2, Cry1F) and coleopteran (Cry3Bb1, Cry34/35Ab1) pests and has exhibited increased effectiveness in reducing WCR larval feeding injury compared to single-trait hybrids [20, 21]. The most recent rootworm-active pyramid granted U.S. registration is SmartStax^®^ PRO, which includes the same rootworm-active proteins as SmartStax^®^ plus the new DvSnf7 dsRNA construct as an additional mode of action [22]. Snf7 (sucrose non-fermenting 7) is a class E vacuolar sorting protein conserved in many eukaryotes including WCR [23]. This DvSnf7 ortholog [24] is part of the Endosomal Sorting Complex Required for Transport (ESCRT-III) pathway in WCR responsible for internalizing, transporting, sorting, and degrading transmembrane proteins [25, 26]. Knockdown of Snf7 in WCR increases build-up of damaged organelles, misfolded proteins, and toxic compounds that negatively affect cell physiology and homeostasis. This disruption inhibits larval growth and development, leading to mortality in WCR [23].

Field trials assessing SmartStax^®^ PRO root injury conducted at 42 sites across nine Corn Belt states from 2013–2015 indicated that the contribution of DvSnf7 dsRNA in SmartStax^®^ PRO significantly reduced WCR root injury and adult emergence (80–95%) at all sites, even in areas with suspected Cry3Bb1 resistance [27]. Simulation models indicated this reduction in root injury and adult emergence could enhance the durability of Cry3Bb1 and Cry34/35Ab1 and decrease the rate of resistance evolution to SmartStax^®^ PRO relative to SmartStax^®^ [27]. However, continuous cultivation of SmartStax^®^ PRO as a mitigation strategy in areas of Cry3Bb1 and/or Cry34/35Ab1 resistance will likely place increased selection pressure on DvSnf7 dsRNA. Resistance to DvSnf7 dsRNA was recently reported after seven generations of laboratory selection in a field-derived WCR population, indicating that dsRNA resistance alleles exist in field populations and field-evolved resistance to dsRNAs is possible with continuous selection pressure [28].

Life history parameters such as developmental time, longevity, size, weight, fecundity, and egg viability have been assessed in a variety of insect pest populations exposed to Bt and non-Bt crops, providing information on the sublethal effects of dietary exposure to Bt proteins. Key corn insect pests that have been examined include corn earworm (*Helicoverpa zea* (Boddie)) [29–31], fall armyworm (*Spodoptera frugiperda* (J.E. Smith)) [32, 33], and WCR [34, 35]. Exposure of susceptible WCR populations to Cry3Bb1 resulted in prolonged developmental time [36–40], decreased adult longevity [34, 41], reduced adult size [35, 42], and lower fecundity [35]. Similar results have been reported with Cry34/35Ab1, as sublethal exposure led to decreased lifespan [43], reduced adult weight [44], and lower fecundity [43]. Egg viability was not significantly affected by Cry3Bb1 dietary exposure [34] or one generation of Cry34/35Ab1 selection [45]. However, life history traits of WCR populations exposed to rootworm-Bt pyramids or SmartStax^®^ PRO have not been characterized.

Therefore, the objective of this study was to compare life history traits of WCR male/female pairs after various dietary exposure regimens to SmartStax^®^ PRO or non-rootworm Bt corn. Because WCR life history traits can be negatively affected by dietary exposure to Cry3Bb1 or Cry34/35Ab1, the working hypothesis for these experiments was: sublethal effects of SmartStax^®^ PRO exposure will negatively affect WCR life history traits. Collectively, this information will inform efforts to optimize IRM strategies.

## Materials and methods

### Study location and WCR collection

An on-farm research site located in Colfax County, Nebraska, was used for 2018 and 2019 experiments. This stewarded research site was chosen due to its history of high WCR densities, continuous cultivation of rootworm-Bt hybrids, suspected WCR resistance to one or more WCR Bt traits, and enhanced ability to obtain enough WCR from SmartStax^®^ PRO plots for use in experiments. Small-plot field trials with SmartStax^®^ PRO had previously been cultivated in parts of the field; therefore, the WCR population also had some exposure to the DvSnf7 dsRNA trait prior to this study. Field plots of pre-commercial SmartStax^®^ PRO and non-rootworm Bt corn of similar genetic background (hereafter referred to as ‘isoline’) measuring eight rows wide by 9m in length were replicated three times in a randomized complete block design during the 2018 growing season. In 2019, plots were six rows wide by 9m in length and both treatments were replicated four times in a randomized complete block design.

In both seasons, expression of Cry3Bb1 and Cry34/35Ab1 in each SmartStax^®^ PRO plant was confirmed during the vegetative growth stages using QuickStix lateral flow strips (Envirologix, Inc., Portland, ME) in all plots. Testing for expression of DvSnf7 dsRNA requires tissue sampling and laboratory analysis; however, because Cry3Bb1 and DvSnf7 dsRNA are linked on the same T-DNA insertion, positive expression of Cry3Bb1 would also indicate positive expression of DvSnf7 dsRNA [46, 47]. No plants failed to express both Bt proteins in 2018 or 2019 field plots. Corn plants in isoline plots were also tested for negative expression of rootworm-Bt proteins to eliminate possible volunteer plants in plots; no rootworm-Bt expressing plants were identified. Plants within the center four rows of each plot were cut down to approximately 0.75m in height, and one emergence tent (3.7m × 3.7m × 1.9m) was erected over each plot on 23 July 2018 (three total tents per treatment). In 2018, tents were erected about 12 days after initial adult emergence, so the total emergence curve was not recorded. After placement, WCR found within tents were removed so only adults emerging after tent placement were used in experiments. In 2019, plants were cut to a height of 0.75m, and two emergence tents were erected over five rows in each plot on 10 July (eight total tents per treatment) prior to initial adult emergence. WCR adults were aspirated from emergence tents twice weekly during peak emergence and once weekly later in the season using insect collecting chambers inside a SKIL^®^ heavy duty hand-held DC vacuum/aspirator with an attached 18V (1.2Ah) nickel-cadmium battery pack (BioQuip Products, Inc., Rancho Dominguez, CA). WCR were maintained in separate insect collecting chambers by treatment and replication (i.e., emergence tent) for transport back to the laboratory at the University of Nebraska-Lincoln. Adult WCR were collected during six weekly periods in 2018 (23 July-29 August) and seven weekly periods in 2019 (10 July-27 August). Emergence week was defined as each calendar week after tents were initially erected over field plots.

### Greenhouse corn production

The same pre-commercial SmartStax^®^ PRO and isoline corn hybrids planted in Colfax Co. field plots were grown in a University of Nebraska-Lincoln greenhouse to provide fresh ear tissue for WCR dietary exposure experiments from 2018–2020. Individual raised wooden planter boxes (2m × 2m × 1m) were filled with native silty clay loam soil removed from the University of Nebraska Eastern Nebraska Research, Extension and Education Center (UNL-ENREEC, Ithaca, NE). Soil had not been treated with herbicides or insecticides for over 20 years. Prior to planting, each planter box was deep-watered (Ross^®^ Root Feeder Deep Irrigation Feeding System; Easy Gardener Products, Inc., Waco, TX) and fertilizer (75g) was applied (Earl May^®^ Garden & Plant Food Plus fertilizer; 10% N, 10% P, 10% K). In total, six planter boxes of each hybrid were planted biweekly from mid-April until late-July to provide an adequate amount of ear tissue for laboratory experiments. Each planter box contained four rows of corn planted 46cm apart with 15cm seed spacing within rows. Protein expression of Cry3Bb1 and Cry34/35Ab1 in SmartStax^®^ PRO plants was confirmed using QuickStix lateral flow strips at the V4-V6 growth stage [48]. Tassel bags (13.3cm × 14.6cm × 10.8cm × 35.6cm; Seedburo Equipment Company, Des Plaines, IL) were placed over tassels once visible to prevent pollen shed. Silks on each ear were hand-pollinated to prevent cross-pollination between treatments. Corn ears were harvested between the R2-R3 (blister to milk) stage [48] for use in WCR feeding experiments.

### Lifetime dietary exposure experiment

An experiment was conducted to determine if dietary exposure to SmartStax^®^ PRO during larval and adult stages negatively affected female WCR life history traits (i.e., adult longevity and size, lifetime egg production, egg viability). In 2018 and 2019, adult male and female WCR from individual Colfax Co. field plots were maintained together on ear tissue (i.e., cross-section of intact ear containing husk, silk, kernels, and cob; hereafter referred to as ‘adult diet’) from the appropriate treatment in plexiglass cages (28cm^3^) for 3-5d to facilitate mating and initial egg development in females. Individual females from each treatment and field replication were then placed into polystyrene oviposition boxes (5.9cm × 5.9cm × 7.8cm; ShowMan Box, Althor Products, Windsor Locks, CT). WCR females were distinguished from males based on morphological characters at the end of the abdomen [49]. A 2.5cm cross-section of adult diet from the treatment in which the WCR female emerged was placed on a rectangular plastic shelf (4.5cm × 2.5cm × 1.5cm) and attached to the lid of the box with Velcro^®^ to simulate lifetime dietary exposure. A mixed substrate of autoclaved silty clay loam soil (65g) sifted through a U.S.A. Standard Testing Sieve No. 60 (Thermo Fisher Scientific, Waltham, MA) moistened with distilled water (20mL) was provided in the bottom of each polystyrene box as an oviposition site. Adult emergence from field plots determined the number of polystyrene oviposition boxes created for each treatment. The same number of females were fed adult isoline diet in the 2018 and 2019 experiments (n = 87). However, the number of females fed SmartStax^®^ PRO adult diet was different between 2018 (n = 65) and 2019 (n = 18) due to the difference in adult emergence.

Female mortality was recorded every 3-4d when adult diet was replaced. Dead females were placed in plastic vials containing 70% ethanol and adult longevity was calculated as the difference between the field collection date and mortality date. Head capsule widths were measured to the nearest 0.01mm using an AmScope 3.5X-90X Simul-Focal Trinocular Stereo Zoom microscope with an attached 18MP USB3 Camera (United Scope LLC, Irvine, CA). After female mortality was observed, eggs were collected by washing the oviposition substrate through a No. 60 sieve to remove soil; eggs were then washed onto a milk filter (KenAg, Ashland, OH) and counted under a microscope to determine lifetime egg production per female. Eggs were transferred to Petri dishes (Thermo Fisher Scientific, Waltham, MA) containing moistened autoclaved soil (27g soil, 10mL distilled water) and covered with a light layer of dry autoclaved soil. Petri dishes were sealed with Parafilm M (Bemis Company, Inc., Neenah, WI) and subjected to an overwintering regimen (25°C for 1 month, 10°C for 1 month, and 7°C for approximately 4–5 months) to allow pre-diapause development and diapause termination to occur [7, 50].

After diapause termination the following spring, eggs laid by females within the same emergence week were pooled across treatment replications (i.e., all emergence tents per treatment) and washed through a No. 60 sieve to remove soil. From this composite sample, 50 random eggs were transferred to a Petri dish lined with moistened Whatman^™^ Qualitative Filter Paper: Grade 1 Circles (Thermo Fisher Scientific, Waltham, MA) and replicated six times per emergence week for each treatment in which eggs were collected. Once eggs were transferred to the filter paper, each Petri dish was sealed with Parafilm M and held at 25°C. Newly eclosed neonate larvae were counted and removed from each Petri dish daily to calculate egg viability.

### Male dietary exposure experiment

An experiment to characterize the indirect effects of male WCR dietary exposure to SmartStax^®^ PRO or isoline corn prior to mating on female fecundity was conducted in 2018. WCR males collected from individual Colfax Co. field plots were maintained on their respective adult diet in separate plexiglass cages (28cm^3^) for 3-5d to promote reproductive development and sexual maturation. In 2017, a WCR population was collected from the UNL-ENREEC in Saunders Co., NE. Field-collected females were allowed to oviposit in the laboratory and the resultant diapausing eggs (F_1_ generation) were reared to adulthood in 2018 following the standard University of Nebraska-Lincoln rearing protocol [Appendix II in 51] to obtain virgin females. This population was chosen for use in this experiment because of its relative susceptibility to Bt proteins [7, 12, 52], lack of previous exposure to DvSnf7 dsRNA, and therefore, decreased likelihood of existing fitness effects from prior selection with Bt proteins or RNAi technology.

Emerging adults were aspirated from the rearing cage daily and virgin females were maintained in 28cm^3^ plexiglass cages by date with isoline diet for 3d prior to use in this experiment. The Saunders Co. population emergence curve was timed to coincide with the natural Colfax Co. emergence curve to have WCR of similar ages placed in polystyrene oviposition boxes. One field-collected male and one teneral laboratory-reared female were placed in a polystyrene oviposition box with oviposition substrate and adult isoline diet as previously described. In total, 26 male/female pairs were established with males emerging from SmartStax^®^ PRO plots across five emergence weeks, and 78 male/female pairs were created with males emerging from isoline plots across six emergence weeks. Life history parameters including male longevity, male head capsule width, lifetime egg production, and egg viability were characterized using the procedures outlined above.

### Adult dietary exposure experiment

An experiment was conducted to determine if dietary exposure to SmartStax^®^ PRO only during the adult stage negatively affected male and female WCR life history traits (i.e., adult longevity and size, lifetime egg production, egg viability). In 2019, WCR from individual Colfax Co. field plots were placed in separate plexiglass cages (28cm^3^) and male/female pairs from each treatment and replication were transferred to polystyrene oviposition boxes containing oviposition substrate within 24h after collection. Male/female pairs from isoline plots were fed SmartStax^®^ PRO or isoline adult diet as previously described to simulate different adult exposures (n = 80 per treatment). Few WCR emerged from SmartStax^®^ PRO field plots in 2019; therefore, the reciprocal dietary exposure regimens were not conducted. Life history parameters including adult longevity, adult head capsule width, lifetime egg production, and egg viability were characterized using the procedures outlined above.

The adult dietary exposure experiment was repeated in 2020. Extra adult WCR collected from Colfax Co. isoline plots by emergence week in 2019 were placed into a single 28cm^3^ plexiglass cage for egg collection; F_1_ eggs were collected from six of seven emergence weeks for use when repeating the experiment. This enabled WCR from the same site with a similar genetic background to be used for each experiment. F_1_ neonates from each emergence week were reared on non-Bt corn (Reid’s Yellow Dent Corn; R.H. Shumway’s Seed Company, Randolph, WI) to adulthood on a staggered schedule following the standard University of Nebraska-Lincoln rearing protocol [Appendix II in 51]. Male/female pairs from each treatment and emergence week were transferred to polystyrene oviposition boxes containing oviposition substrate within 24h after emergence and fed SmartStax^®^ PRO or isoline adult diet. Within each emergence week for both treatments, 12 male/female pairs were established (total n = 72 per treatment). Life history parameters (adult longevity, adult head capsule width, lifetime egg production, and egg viability) were characterized as previously described.

### Single-plant larval bioassays

Single-plant bioassays [5] were conducted with F_1_ neonate progeny obtained from adults collected from isoline emergence tents at the Colfax Co. field site and a trap crop at UNL-ENREEC in Saunders Co. to determine the susceptibility of these WCR populations to Cry3Bb1 and Cry3Bb1 + Cry34/35Ab1 (SmartStax^®^). The WCR population at the Saunders Co. site served as a field control as it has had minimal exposure to Bt traits and has consistently tested as susceptible to Bt proteins in past assays [7, 12, 52]. Bioassays were conducted during the spring and summer of 2019 and 2020 (i.e., the year following collection). The procedures used to collect eggs, maintain adults, and temperature regimens used to facilitate egg diapause and post-diapause development are described in Wangila et al. [7]. Four diapausing WCR colonies (Butler Co., NE [collected in 1990]; Potter Co., SD [1995]; Finney Co., KS [2000]; Centre Co., PA [2000]) continuously reared and maintained at the USDA-ARS North Central Agricultural Laboratory were used as lab controls in 2019 and 2020 bioassays. These colonies remain susceptible to rootworm-Bt proteins because they were collected prior to the commercialization of Bt proteins and wild-type genes have not been introduced since initial collection. Neonate WCR larvae from each population or colony were assayed on three corn hybrids without seed treatments: 1) DKC 66–87 GENVT2P (non-rootworm Bt), 2) DKC 64–69 GENVT3P (Cry3Bb1), and DKC 64–34 GENSS (Cry3Bb1 + Cry34/35Ab1) according to the methods outlined in Reinders et al. [12, 52]. In brief, 12 plants of each corn hybrid were grown to the V4-V5 growth stage [48] in 1L plastic pots (Johnson Paper & Supply Company, Minneapolis, MN) under greenhouse conditions. Each plant was infested with 12 neonate WCR larvae and maintained at 24°C with a 14h:10h (L:D) photoperiod for 17 days to promote larval feeding and development prior to collection of larval survivors using a Berlese funnel.

### Data analysis

All data were analyzed using SAS 9.4 software [53]. Statistical significance was reported at α = 0.05 for all analyses. Separate analyses were performed for each year an experiment was conducted.

#### Adult emergence summary

Temporal WCR adult emergence patterns from SmartStax^®^ PRO and isoline field plots during 2019 (only year of study full emergence curve obtained) were summarized by adding temporal emergence from all field replicates (i.e., emergence tents) and calculating the aggregate number of days to 50% emergence per treatment by sex. Formal statistical comparison of temporal emergence among treatment × sex combinations was not possible because emergence from SmartStax^®^ PRO was too low.

#### Life history parameter analysis

Prior to conducting the final analyses for each life history trait, an initial analysis was conducted to test for variation among field replications (longevity, head capsule width, egg production) or emergence week (egg viability) within each dietary exposure experiment. A generalized linear mixed model (GLMM) or linear mixed model (LMM) (implemented using PROC GLIMMIX [53]) was used to analyze the effect of lifetime or adult diet treatment on adult longevity (GLMM, negative binomial distribution [54, 55]), adult head capsule width (LMM, normal distribution), lifetime egg production (GLMM, negative binomial distribution), or egg viability (GLMM, beta distribution [54, 56]) in all experiments. The initial model included diet treatment and field replication or diet treatment and emergence week as fixed factors and the interaction of diet treatment and field replication or diet treatment and emergence week as a random factor to ensure the denominator degrees of freedom accurately represented the experimental design. Because the 2020 adult dietary exposure experiment did not include field replications, the initial model for this experiment included adult diet treatment and emergence week as fixed factors and the interaction of adult diet treatment and emergence week as a random factor. The main effect of field replication or emergence week was not significant in any analyses and was therefore removed from the model.

In the final analyses, a GLMM or LMM (implemented using PROC GLIMMIX [53]) with a one-way treatment structure was used to analyze the effect of lifetime or adult diet treatment on adult longevity (GLMM, negative binomial distribution), adult head capsule width (LMM, normal distribution), lifetime egg production (GLMM, negative binomial distribution), or egg viability (GLMM, beta distribution). The model included diet treatment as a fixed factor and the interaction of diet treatment and field replication or emergence week as a random factor based upon the experimental design. The LSMEANS statement with the SLICEDIFF option was used to identify significant differences in life history parameters between diet treatments. A post hoc analysis was not conducted because only two group means were analyzed. Results from diet treatment LSMEANS and associated standard errors are reported in this manuscript.

#### Single-plant bioassay analysis

Within a population, proportional survival on each individual corn plant was calculated by dividing the number of larval survivors by 12 (i.e., number of larvae infested per plant). A GLMM (implemented using PROC GLIMMIX [53]) following a binomial distribution with a logit link function [54, 55] was then used to evaluate proportional survival on each corn hybrid recorded in bioassays. Data from the four lab control colonies were pooled within a bioassay year to create a composite sample due to the similar response among populations on each Bt hybrid (S1 Table). Bioassay results from 2019 and 2020 were analyzed separately. The model included population, corn hybrid, and the population by corn hybrid interaction as fixed factors. Observation nested within the population by corn hybrid interaction was included in the model as a random factor to control for an overdispersion of variance [55]. Model fit was evaluated based on generalized chi-square/df value (i.e., approximately 1) and conditional residual plots. Tukey’s multiplicity adjustment was used to control for type I error rates when making pairwise comparisons.

## Results

### Adult emergence in field plots

Adult WCR were present in SmartStax^®^ PRO and isoline tents during all emergence periods from 23 July-29 August 2018. In 2019, WCR were present in isoline tents during all weekly emergence periods (10 July-27 August 2019) but were only present in SmartStax^®^ PRO tents during five emergence periods from 24 July-27 August 2019. During each year, WCR survival from SmartStax^®^ PRO plots was very low compared to isoline plots (Table 1). The aggregate number of days after tent placement to 50% adult emergence varied among treatments (isoline: males 14d, females 18d; SmartStax^®^ PRO: males 25d, females 33d; S1 Fig).

**Table 1:** Stand count and adult western corn rootworm emergence data for SmartStax^®^ PRO and isoline field plots.

Year	Treatment	Total Plants ^a^	Total WCR Emergence ^b^	# WCR/Plant	% Reduction ^c^
2018	SmartStax^®^ PRO	266	125	0.47	97.1%
	Isoline	280	4,406	15.7	-
2019	SmartStax^®^ PRO	606	27	0.04	99.7%
	Isoline	506	7,846	15.5	-

^a^Total plants sampled from three tents in 2018 and eight tents in 2019. Each plot had one emergence tent in 2018 and two emergence tents in 2019.

^b^Total number of western corn rootworm adults collected from all emergence tents per treatment.

^c^Percent reduction in adult emergence compared to isoline due to SmartStax^®^ PRO larval exposure.

### Lifetime dietary exposure experiment

**Fig 1:**
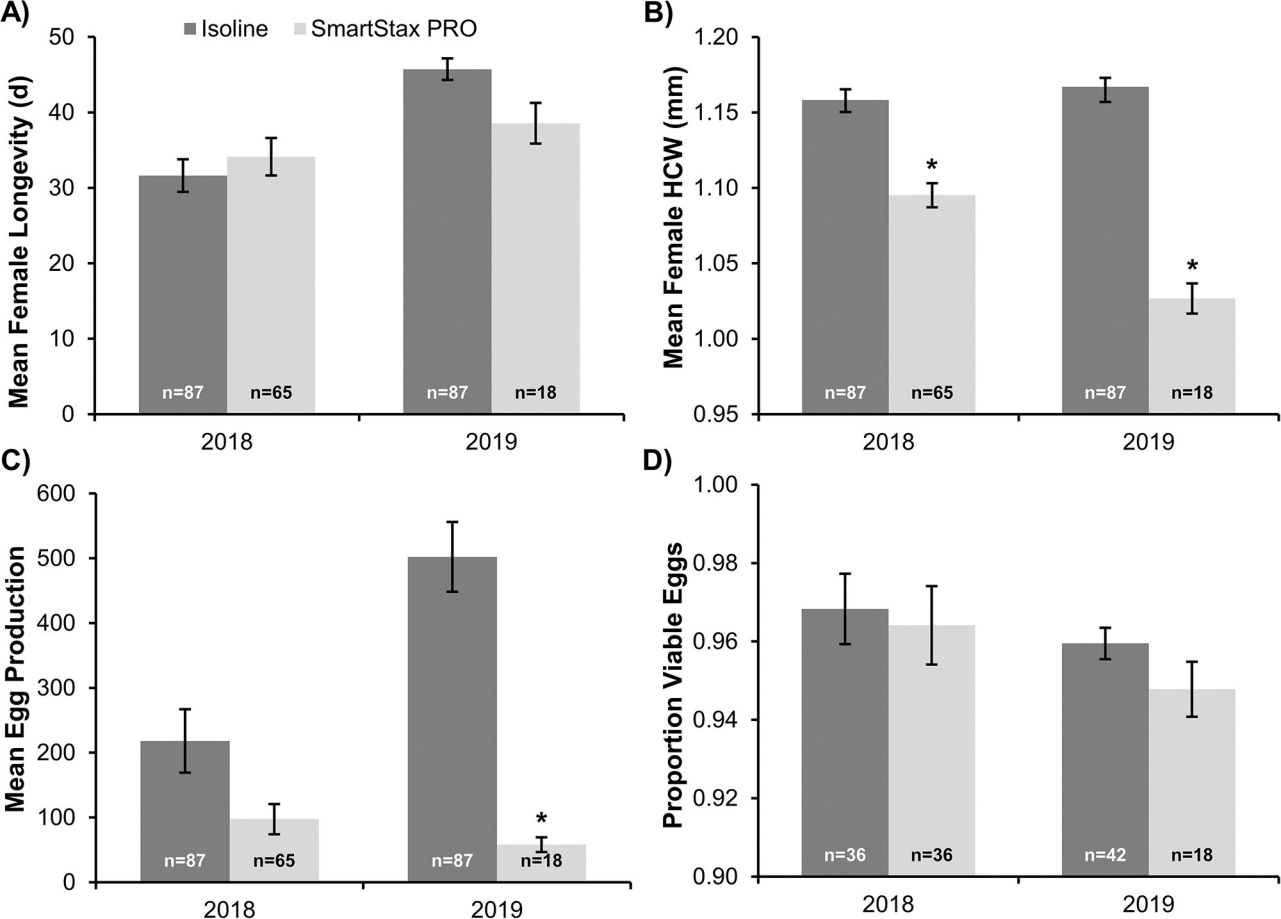
Life history parameters characterized during lifetime dietary exposure experiments in 2018 and 2019. A) Mean female longevity (days, d), B) Mean female head capsule width (HCW; mm), C) Mean egg production per female, and D) Proportion of viable F_1_ eggs. Data from 2018 and 2019 experiments were analyzed separately. Individual bars represent the mean ± standard error and include the number of male/female pairs (n) established for each lifetime diet treatment. An asterisk indicates significant differences between lifetime diet treatments within years (*P*<0.05).

Mean WCR adult female longevity was not significantly affected by lifetime diet treatment in 2018 (*F*_1,4_ = 0.58, *P* = 0.4892) or 2019 (*F*_1,5_ = 4.97, *P* = 0.0763) experiments (Fig 1A), but lifetime SmartStax^®^ PRO exposure did significantly reduce mean female head capsule width (HCW) by approximately 0.06mm in 2018 (*F*_1,4_ = 33.88, *P* = 0.0043) and approximately 0.14mm in 2019 (*F*_1,5_ = 108.25, *P* = 0.0001) (Fig 1B). A significant 88.5% decrease in egg production after lifetime SmartStax^®^ PRO dietary exposure was observed in 2019 when compared to the isoline treatment (*F*_1,5_ = 92.98, *P* = 0.0002) (Fig 1C). A similar trend was observed in 2018; i.e., 55% reduction in egg production after dietary exposure to SmartStax^®^ PRO, but this difference between treatments was not significant (*F*_1,4_ = 5.99, *P* = 0.0706). F_1_ egg viability was relatively high (≥ 95%) and not significantly affected by lifetime diet treatment in 2018 (*F*_1,10_ = 0.10, *P* = 0.7591) or 2019 (*F*_1,8_ = 2.66, *P* = 0.1413) experiments (Fig 1D).

### Male dietary exposure experiment

**Fig 2:**
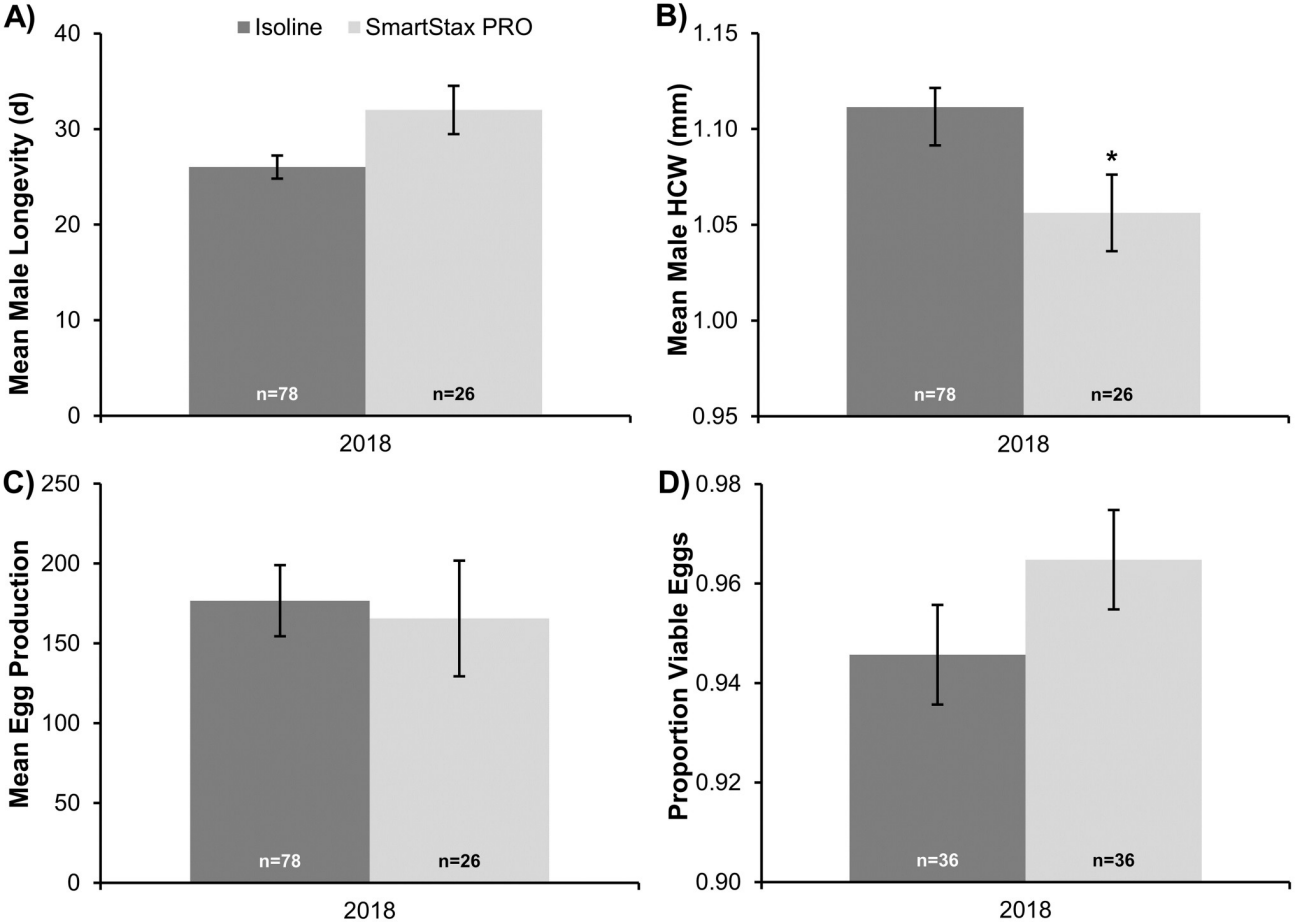
Life history parameters characterized during the male dietary exposure experiment conducted in 2018. A) Mean male longevity (days, d), B) Mean male head capsule width (HCW; mm), C) Mean egg production per UNL-ENREEC female by male diet treatment prior to mating, and D) Proportion of viable F_1_ eggs from UNL-ENREEC females by male diet treatment prior to mating. Individual bars represent the mean ± standard error and include the number of male/female pairs (n) established for each male diet treatment. An asterisk indicates significant differences between diet treatments (*P*<0.05).

Mean male longevity was not significantly affected by diet treatment (*F*_1,4_ = 5.09, *P* = 0.0871) (Fig 2A), but a significant decrease in mean male head capsule width of approximately 0.05mm was associated with larval SmartStax^®^ PRO dietary exposure (*F*_1,4_ = 7.79, *P* = 0.0492) (Fig 2B). Male exposure to SmartStax^®^ PRO or isoline diet prior to mating did not significantly affect lifetime egg production of F_1_ UNL-ENREEC females (*F*_1,4_ = 0.07, *P* = 0.8084) (Fig 2C) or F_1_ egg viability (*F*_1,9_ = 1.74, *P* = 0.2195) (Fig 2D).

### Adult dietary exposure experiment

**Fig 3:**
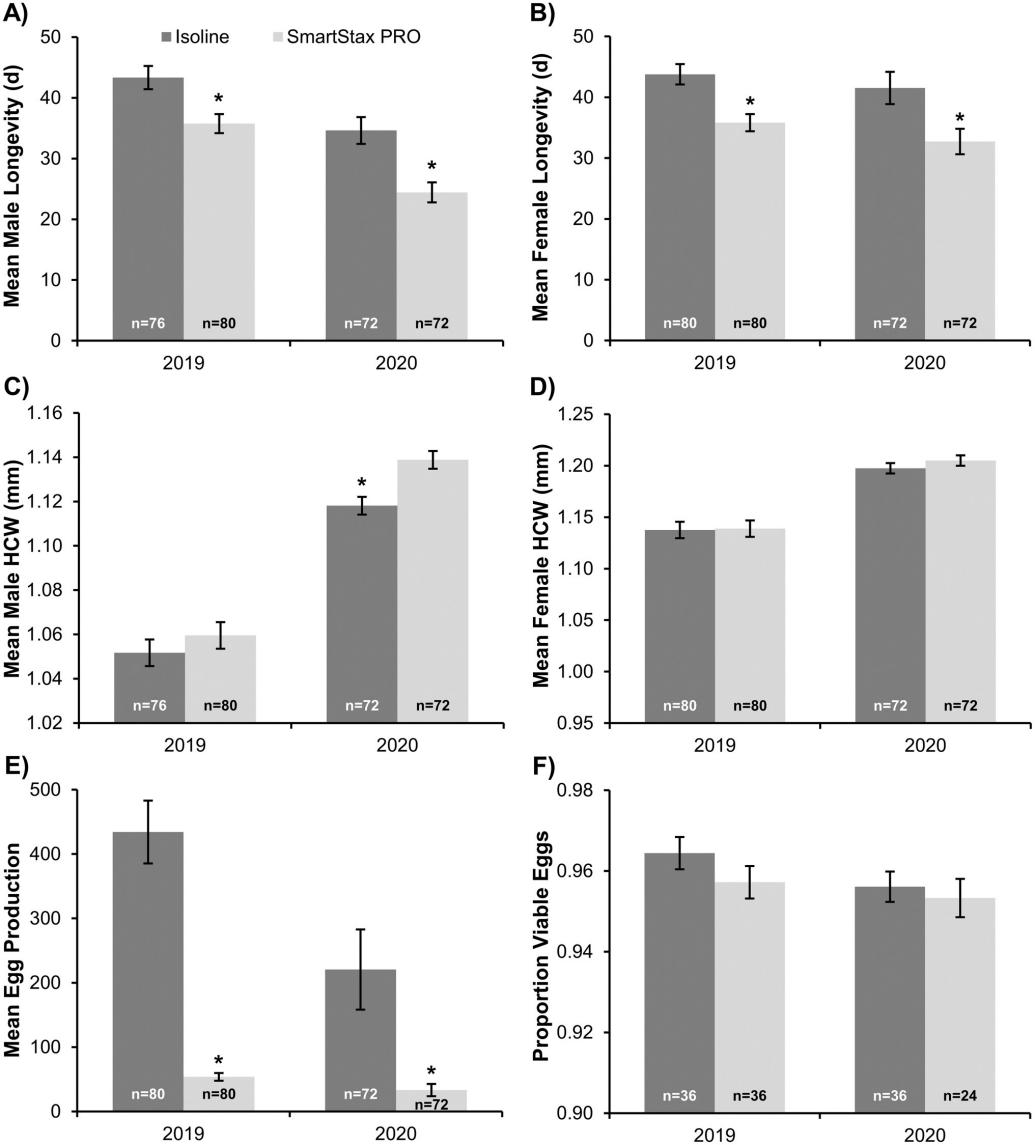
Life history parameters characterized during adult dietary exposure experiments in 2019 and 2020. A) Mean male longevity (days, d), B) Mean female longevity (days, d), C) Mean male head capsule width (HCW; mm), D) Mean female head capsule width (HCW; mm), E) Mean egg production per female, and F) Proportion of viable F_1_ eggs. Data from 2019 and 2020 experiments were analyzed separately. Individual bars represent the mean ± standard error and include the number of male/female pairs (n) established for each adult diet treatment. An asterisk indicates significant differences between adult diet treatments within years (*P*<0.05).

Adult SmartStax^®^ PRO dietary exposure significantly decreased mean male (2019: *F*_1,6_ = 9.55, *P* = 0.0214; 2020: *F*_1,10_ = 11.48, *P* = 0.0069) and female (2019: *F*_1,6_ = 13.52, *P* = 0.0104; 2020: *F*_1,10_ = 6.94, *P* = 0.0250) longevity by 7-10d (Fig 3A and 3B). Adult feeding on SmartStax^®^ PRO or isoline diet did not significantly affect mean male head capsule width in the 2019 experiment (*F*_1,6_ = 0.92, *P* = 0.3754). However, male head capsule width was significantly larger (0.02mm) when fed SmartStax^®^ PRO than isoline diet in the 2020 experiment (*F*_1,10_ = 8.58, *P* = 0.0151) (Fig 3C). Mean head capsule width of adult female WCR was not significantly different between adult diet treatments in 2019 (*F*_1,6_ = 0.02, *P* = 0.9052) and 2020 (*F*_1,10_ = 1.25, *P* = 0.2896) experiments (Fig 3D). Adult SmartStax^®^ PRO dietary exposure resulted in a significant decrease in mean egg production by approximately 85–88% in 2019 (*F*_1,6_ = 171.21, *P*<0.0001) and 2020 (*F*_1,10_ = 22.33, *P* = 0.0008) experiments (Fig 3E). Viability of F_1_ eggs was relatively high (≥ 95%) and was not significantly affected by adult diet treatment in the 2019 (*F*_1,10_ = 1.41, *P* = 0.2618) or 2020 (*F*_1,8_ = 0.21, *P* = 0.6554) experiments (Fig 3F).

### Single-plant larval bioassays

**Fig 4:**
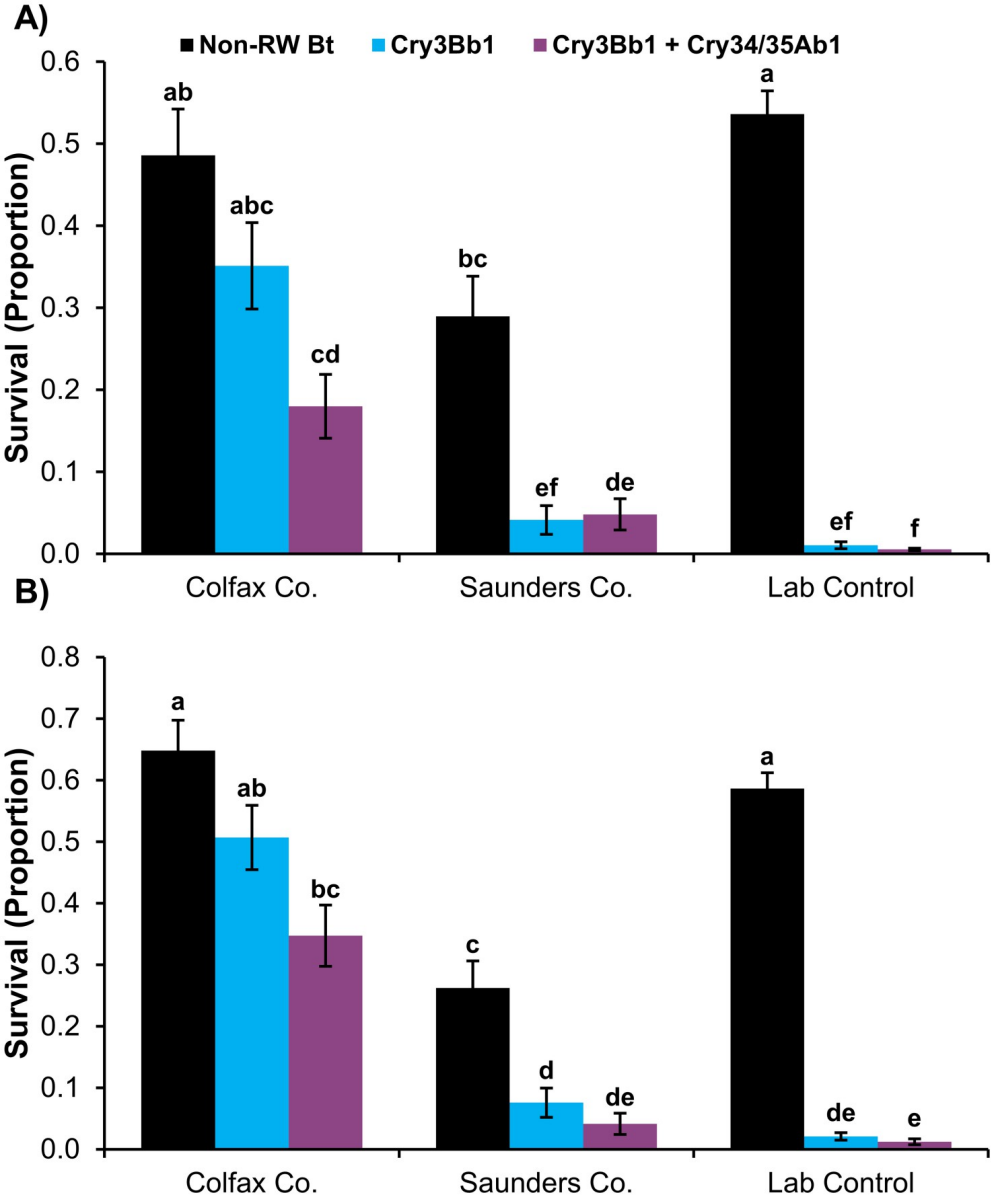
Mean larval survival (± SE) of field-collected WCR populations and susceptible lab control colonies used in plant-based bioassays. A) 2019 bioassays and B) 2020 bioassays. Non-RW Bt = non-rootworm active Bt corn; Cry3Bb1 + Cry34/35Ab1 = SmartStax^®^. Bars with the same letter were not significantly different (GLMM, *P*>0.05).

A significant interaction between population and corn hybrid for proportional survival occurred in both 2019 and 2020 bioassays (2019: *F*_4,207_ = 20.32, *P*<0.0001; 2020: *F*_4,207_ = 25.46, *P*<0.0001) (Fig 4). Mean larval survival on the Cry3Bb1 and non-rootworm Bt hybrid was not significantly different in 2019 and 2020 Colfax Co. bioassays, but mean survival on SmartStax^®^ (Cry3Bb1 + Cry34/35Ab1) was significantly lower than the non-rootworm Bt hybrid in both bioassay years. The Colfax Co. population exhibited significantly higher mean survival on the Cry3Bb1 and SmartStax^®^ hybrids compared to the Saunders Co. population (Cry3Bb1: 2019 and 2020; SmartStax^®^: 2020) and lab control colonies (2019 and 2020), which had very low mean survival on each Bt hybrid. The Saunders Co. population and lab control colonies also exhibited significantly lower mean survival on both rootworm-Bt hybrids compared to the non-rootworm Bt hybrid in both bioassay years.

## Discussion

The large reduction in adult emergence (97.1–99.7%) observed after WCR larval exposure to SmartStax^®^ PRO falls within the range previously reported in SmartStax^®^ PRO field trials conducted across the U.S. Corn Belt [27]. The reduction observed in 2018 (97.1%) was probably conservative as initial emergence was not included in the percent reduction calculation. This high larval mortality and the significant reductions in adult longevity, size, and egg production of SmartStax^®^ PRO survivors documented in this study collectively support the working hypothesis that WCR dietary exposure to SmartStax^®^ PRO will negatively affect WCR life history traits and fitness. All of these factors may have direct or indirect effects on WCR population dynamics and potential resistance evolution to the pyramid.

The reduced egg production of females exposed to a lifetime or adult SmartStax^®^ PRO diet appears to be the key life history parameter impacting adult fitness in this study. Head capsule width is a relatively stable phenotypic parameter [57] commonly used to assess fitness in WCR adults, as there is often a correlation between adult female size and lifetime fecundity [58]. Sublethal exposure to Bt corn [42, 57, 59] or larval crowding and associated food availability (i.e., density-dependent effects) [58] can significantly decrease adult head capsule width. Because of the significant density reduction in SmartStax^®^ PRO plots, crowding and food availability should not have been limiting factors in this study. In addition, size differences would not explain the decrease in egg production of WCR females exposed to SmartStax^®^ PRO in the adult exposure experiment (Fig 3E). Therefore, dietary exposure to one or more traits in SmartStax^®^ PRO likely contributed directly or indirectly to the reduced egg production observed in this study. Egg viability was not significantly affected by lifetime or adult SmartStax^®^ PRO dietary exposure in this study; similar results have been reported in susceptible WCR populations exposed to Cry3Bb1 [34] or Cry34/35Ab1 [45] and WCR resistant to Cry34/35Ab1 [60]. Although egg fertility remained high, fecundity was greatly reduced because of low egg production after sublethal dietary exposure to SmartStax^®^ PRO.

It is unclear if the significant decrease in adult male (Fig 3A) and female (Fig 3B) longevity associated with adult SmartStax^®^ PRO exposure may reduce the mating and oviposition periods under field conditions. Previous research indicates that male mating ability and the number of mating attempts declines with age [61]. Male WCR require 5-7d of post-emergence development to reach sexual maturity and most males mate with newly emerged females upon reaching sexual maturity [62]. Most additional mating attempts occur within 10d after initial mating and decline in frequency from 11-20d after initial mating [61]. Therefore, except for mating attempts late in the male lifespan, the approximately 7-10d reduction in male lifespan observed in this study may not be sufficient to reduce the majority of mating attempts under field conditions. In WCR females, the pre-ovipositional period can range from 6d [63] to 21d [64] and oviposition can occur for up to 60d after successful mating [63, 65, 66]. Previous studies indicate that WCR oviposition peaks around 10-15d into the oviposition period and declines with time [65, 66]. The shortened female lifespan may decrease lifetime egg production but may not affect the peak oviposition period. Additional research is needed to understand the potential impacts of reduced adult WCR longevity identified in this study on the population density present in the subsequent growing season.

The asynchronous adult WCR emergence patterns of susceptible populations often associated with later peak emergence from rootworm-Bt corn than the non-rootworm Bt refuge [36–40] and WCR pre-copulatory behavior provide opportunities for discriminatory mate selection, which could play an important role in mating dynamics within large cornfields. Emergence curves from SmartStax^®^ PRO and isoline available from 2019 (S1 Fig) when survival on SmartStax^®^ PRO was very low (Table 1) were consistent with the asynchronous emergence patterns typically reported for WCR populations susceptible to Bt proteins. Depending on the larval density (crowding) present, WCR emerging from non-rootworm Bt refuge plants could be larger or closer in size to WCR emerging from SmartStax^®^ PRO plants. In this study, because field-collected females and males from the isoline treatment were significantly larger than those collected from the SmartStax^®^ PRO treatment, in a field environment, these size differences may contribute to mate-choice and mating success. Large males tend to initiate mating earlier and mate more frequently than small males [67] and larger females may refuse smaller males [67, 68]. Positive relationships between female size and the number of male mating attempts [69] have also been reported. However, it should be noted that lifetime egg production was not affected when smaller males emerging from SmartStax^®^ PRO plants were mated with refuge females (Fig 2C), suggesting the sublethal effect of SmartStax^®^ PRO on male fertility was minimal. The emergence patterns and potential pre-copulatory behavioral barriers associated with SmartStax^®^ PRO need further investigation but results from this study suggest that unintended assortative mating between adults emerging from SmartStax^®^ PRO plants may occur, especially later in the season. These factors could accelerate resistance evolution [15].

The potential contribution of each rootworm-active trait expressed in SmartStax^®^ PRO to the decrease in fitness observed in this study is currently unclear. Bioassay results indicate that Cry3Bb1 and/or Cry34/35Ab1 Bt resistance alleles are fairly common in the WCR population at the Colfax Co. location (Fig 4), suggesting that a mixture of resistant and susceptible individuals is present. Although reduced fecundity has been observed when susceptible WCR populations fed on Cry3Bb1 [35] or Cry34/35Ab1 [43], few fitness costs have been associated with laboratory-selected or field-evolved resistance to Cry3Bb1 in WCR populations; this includes potential effects on fecundity (reviewed in [70]). A single study has documented potential fitness costs associated with dietary exposure of WCR resistant to Cry34/35Ab1 [60]. Cry34/35Ab1-resistant WCR populations exhibited reduced size, longevity, and lifetime egg production after dietary exposure to Cry34/35Ab1 and also reverted back to susceptibility six generations after removal from selection with Cry34/35Ab1 [60].

To date, no information is available on the possible impact of resistance to DvSnf7 dsRNA on WCR fitness. Some negative effects on fecundity were reported when southern corn rootworm (SCR), *Diabrotica undecimpunctata howardi* Barber, adults were exposed to the estimated LC_50_ of Snf7 dsRNA overlaid on artificial diet [71, 72]. However, it is important to note that the SCR is more sensitive to Snf7 dsRNA than the WCR [71]. Sublethal exposure of WCR larvae and adults will occur in cornfields where SmartStax^®^ PRO is planted because DvSnf7 dsRNA is expressed in root and above-ground tissues in corn event MON 87411 [73]. However, the relatively low DvSnf7 dsRNA expression levels [73] and lower sensitivity of adults than larvae to Snf7 dsRNA [71] may result in negligible fitness effects on adult WCR. WCR population responses to SmartStax^®^ PRO dietary exposure could be variable depending on which traits contribute to fitness costs and the frequency of resistant WCR individuals in a specific population. Therefore, a broader dataset is needed to sort out fitness cause and effects when SmartStax^®^ PRO is deployed in the field.

This study provides an example of a WCR population that exhibited complete resistance to Cry3Bb1 and incomplete resistance to SmartStax^®^ but still incurred significant reductions in larval survival and adult fitness (i.e., female size, longevity, egg production; male size, longevity) after dietary exposure to SmartStax^®^ PRO. Complete resistance is defined as no significant difference in bioassay survival between WCR reared on Bt and isoline hybrids [52, 74] while WCR populations with incomplete resistance exhibit a significant difference in bioassay survival between Bt and isoline hybrids [52, 75]. In each case, survival on Bt is significantly greater than lab control colonies. Complete resistance to Cry3Bb1 indicates a very high frequency of Cry3Bb1-resistant individuals in the Colfax Co. population. To date, there is no published example of a WCR population with complete resistance to Cry3Bb1 where fecundity is adversely affected by dietary exposure to Cry3Bb1 [70, 74]; therefore, Cry3Bb1 may be contributing little to the reduction in fitness observed in this study. Incomplete resistance to SmartStax^®^ suggests that susceptible or resistant WCR receiving sublethal exposure to Cry34/35Ab1 may have played a role in the fecundity reduction [43, 60]. Because SmartStax^®^ contains the same Bt proteins as SmartStax^®^ PRO, but WCR adult fitness data have not been reported for SmartStax^®^, additional studies are needed to determine if reductions in WCR fecundity after dietary exposure are similar between the pyramids. This comparison would also help identify any possible contribution of DvSnf7 dsRNA to the reduced adult fitness reported in this study.

The great reduction in survival to adulthood and the significant sublethal effects of SmartStax^®^ PRO dietary exposure on WCR fitness observed in this study collectively suggest that a significant decrease in local population growth may occur when SmartStax^®^ PRO is deployed in the field. The SmartStax^®^ PRO pyramid will be sold as a 95:5 seed blend, facilitating ingestion of transgenic tissue by almost all WCR present in a field at some point during their life cycle. Although the feeding duration necessary to cause detrimental effects on fitness parameters is currently unknown, results from the adult exposure experiment suggest that the reproductive capability of individuals emerging from refuge plants or migrating into fields planted with SmartStax^®^ PRO could be significantly impacted and contribute to negative effects on population dynamics. This suggests that SmartStax^®^ PRO will be a good tool to help mitigate WCR Bt resistance by greatly reducing WCR densities and fecundity of survivors. Modeling exercises are needed to determine how SmartStax^®^ PRO dietary exposure may impact WCR population growth parameters and the potential for resistance evolution under various scenarios to inform resistance management strategies. In continuous corn production areas of the U.S. Corn Belt, WCR plant-incorporated protectant options are currently limited and metapopulations within an area will continuously be exposed to existing Bt protein combinations. Genetic mixing within the metapopulation due to the movement of WCR resistance alleles in the landscape [12, 76] and the persistence of Bt resistance for multiple generations after removal of selection pressure [77–80] may enable WCR resistance to SmartStax^®^ PRO or other technologies to be maintained in an area over time.

The high WCR larval mortality associated with SmartStax^®^ PRO cultivation may allow growers to better manage WCR larval injury in continuous corn [27] and potentially incorporate alternative management tactics in the subsequent growing season (e.g., hybrid not expressing rootworm traits plus soil insecticide or high-rate seed treatment), prolonging the durability of this new product. Because the WCR is highly adaptable to management tactics [81; reviewed in 70, 82], deploying SmartStax^®^ PRO within an integrated pest management framework will be critical to reduce selection and slow WCR resistance evolution to RNAi technology. A more holistic approach to WCR management that incorporates multiple tactics and rotation of tactics is needed to preserve WCR susceptibility to SmartStax^®^ PRO and future plant-incorporated protectants [15, 27, 83, 84].

## Supporting information

S1 Table Mean proportional survival (± SE) of susceptible lab control colonies. (A) Cry3Bb1 in 2019 bioassays, (B) Cry3Bb1 in 2020 bioassays, (C) Cry3Bb1 + Cry34/35Ab1 in 2019 bioassays, and (D) Cry3Bb1 + Cry34/35Ab1 in 2020 bioassays. Within hybrids and years, no significant differences in mean survival among colonies were documented (GLMM, binomial distribution; P > 0.05).
(DOCX)

S1 Fig Male and female western corn rootworm adult emergence curves from SmartStax^®^ PRO and isoline field plots, 2019. Each light gray bar represents the numerical total of male or female western corn rootworms collected from the eight emergence tents placed over replicated field plots of each treatment. The solid blue and dashed orange vertical lines denote the number of days after tent placement when 50% adult emergence occurred for each sex and treatment. WCR were collected twice weekly during the first four emergence periods and once weekly during the last three emergence periods.
(TIFF)
