# Supplementary material for: Characterizing the sublethal effects of SmartStax PRO dietary exposure on life history traits of the western corn rootworm, Diabrotica virgifera virgifera LeConte
Source: PLoS One. 2022 May 25;17(5):e0268902. doi: 10.1371/journal.pone.0268902 (PMC9132300; doi:10.1371/journal.pone.0268902)
Supplement: S1 Table — (A) Cry3Bb1 in 2019 bioassays, (B) Cry3Bb1 in 2020 bioassays, (C) Cry3Bb1 + Cry34/35Ab1 in 2019 bioassays, and (D) Cry3Bb1 + Cry34/35Ab1 in 2020 bioassays. Within hybrids and years, no significant differences in mean survival among colonies were documented (GLMM, binomial distribution; P > 0.05). (DOCX) [file pone.0268902.s001.docx]

**S1 Table. Mean proportional survival (± SE) of susceptible lab control colonies.** (A) Cry3Bb1 in 2019 bioassays, (B) Cry3Bb1 in 2020 bioassays, (C) Cry3Bb1+ Cry34/35Ab1 in 2019 bioassays, and (D) Cry3Bb1 + Cry34/35Ab1 in 2020 bioassays. Within hybrids and years, no significant differences in mean survival among colonies were documented (GLMM, binomial distribution; *P*>0.05).
