# Supplementary figures and images for: Characterizing the sublethal effects of SmartStax PRO dietary exposure on life history traits of the western corn rootworm, Diabrotica virgifera virgifera LeConte
Source: PLoS One. 2022 May 25;17(5):e0268902. doi: 10.1371/journal.pone.0268902 (PMC9132300; doi:10.1371/journal.pone.0268902)

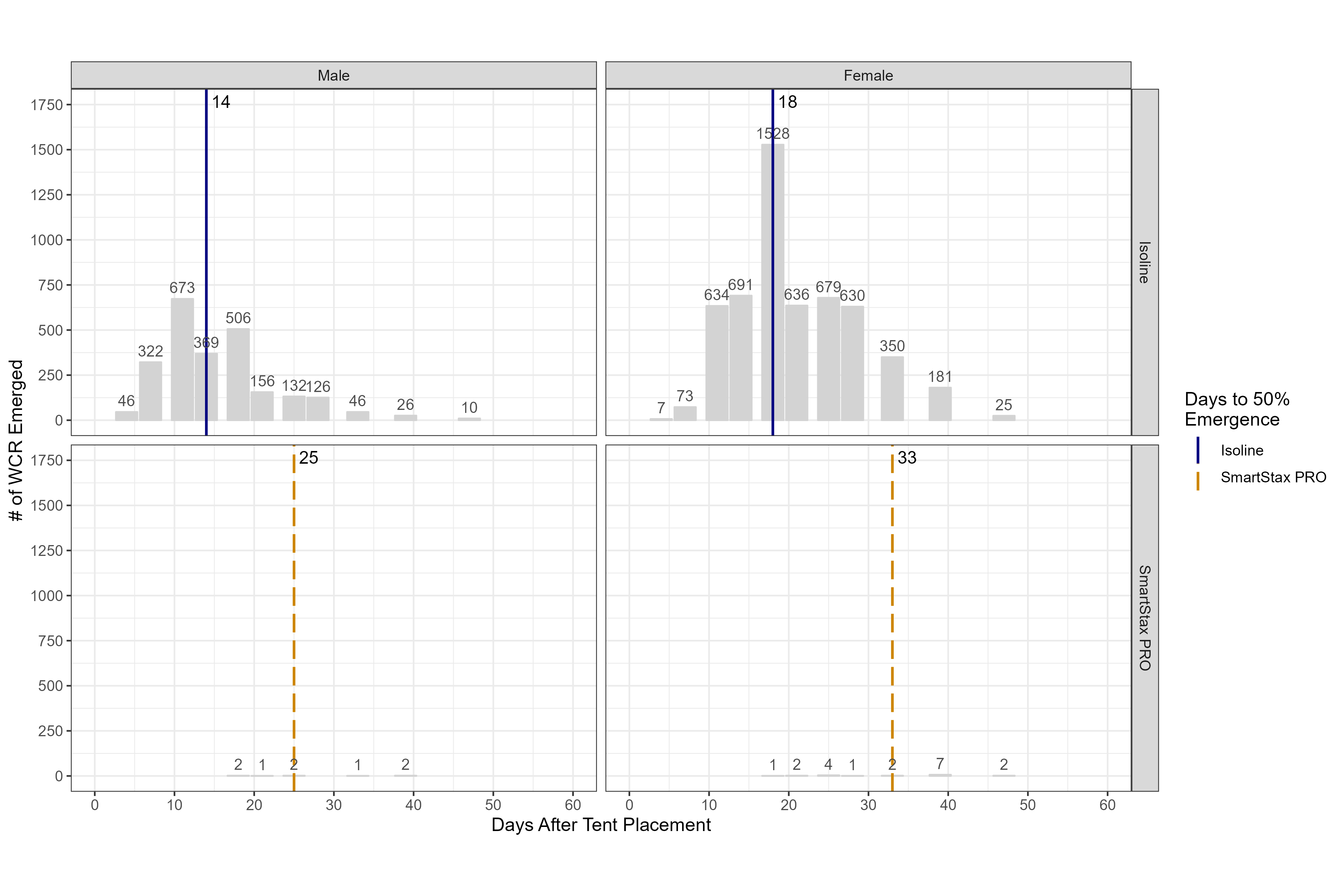

Supplement: S1 Fig — Each light gray bar represents the numerical total of male or female western corn rootworms collected from the eight emergence tents placed over replicated field plots of each treatment. The solid blue and dashed orange vertical lines denote the number of days after tent placement when 50% adult emergence occurred for each sex and treatment. WCR were collected twice weekly during the first four emergence periods and once weekly during the last three emergence periods. (TIFF) [file pone.0268902.s002.tiff]
